# Effects of Chest Physiotherapy on Reducing Dyspnea and Enhancing Functional Independence and Quality of Life in Multilobar Pneumonia: A Case Report

**DOI:** 10.7759/cureus.70868

**Published:** 2024-10-04

**Authors:** Pinky D Israni, Lajwanti Lalwani, Samruddhi Aherrao

**Affiliations:** 1 Cardiovascular and Respiratory Physiotherapy, Ravi Nair Physiotherapy College, Datta Meghe Institute of Higher Education & Research, Wardha, IND

**Keywords:** airway clearance techniques, bronchopneumonia, chest physiotherapy, early mobility, pulmonary rehabilitation

## Abstract

Bronchopneumonia is characterized by inflammation of the lungs, predominantly affecting the bronchioles, whereas lobar pneumonia is a bacterial infection that leads to inflammation primarily in the alveoli and lung lobes. We present the case of a 65-year-old male patient who presented with complaints of breathlessness and cough accompanied by whitish expectoration, ultimately diagnosed with multilobar pneumonia. The patient was subsequently referred for chest physiotherapy to address these complaints. As physiotherapists, we employed a range of treatments, including early mobility, active breathing exercises, and airway clearance techniques. This case underscores the significance of chest physiotherapy for patients with multilobar pneumonia. Outcome measures included the Modified Medical Research Council dyspnea grading scale, the Functional Independence Measure score, and the Numerical Pain Rating Scale. Following the rehabilitation program, improvements were observed in all outcome measures. These findings indicate that a tailored pulmonary rehabilitation program can significantly benefit patients with pneumonia by reducing dyspnea and enhancing functional independence and quality of life.

## Introduction

According to the Global Burden of Diseases, Injuries, and Risk Factors Study (GBD), lower respiratory tract infections, classified as pneumonia or bronchiolitis, are significant contributors to global morbidity and mortality. In 2016, lower respiratory tract infections accounted for approximately 2.38 million deaths, making them the sixth most prevalent cause of death across all age groups [1]. Lobar pneumonia, a bacterial infection, typically leads to inflammation in the lungs, primarily affecting the alveoli and lung lobes [2]. Pneumonia occurs when a pathogen reaches the alveoli and overwhelms the host’s defenses due to the microorganism's virulence or the size of the inoculum [3]. The most common pathogen identified is *Streptococcus pneumoniae*, while *Staphylococcus aureus*, *Haemophilus influenzae*, *Klebsiella *species, and *Legionella pneumophila *occur less frequently [4]. Bacterial endogenous sources include the oropharynx, sinusitis, stomach or tracheal colonization, nasal carriers, and hematogenous spread [5]. In older patients, pneumonia is associated with higher mortality and morbidity rates compared to younger individuals, primarily due to underlying cardiovascular diseases and compromised host defenses [6].

Traditional treatment includes various chest physiotherapy techniques, such as coughing, huffing, postural drainage, and percussion [7]. International guidelines state that the presence of newly discovered or altered infiltrates on a chest radiograph, alongside fever and/or severe respiratory symptoms, serves as the standard criterion for diagnosing pneumonia [8]. Physiotherapy plays a crucial role in the treatment of patients in respiratory intensive care units, aiming to improve overall functional ability and restore physical and respiratory independence, thereby reducing complications associated with bed rest [9]. Early physiotherapy intervention, depending on the severity of pneumonia, can prevent declines in activities of daily living (ADLs) and shorten hospitalization durations for elderly patients in ICU care.

Physiotherapy should be recognized as an effective therapeutic approach that mitigates complications and enhances outcomes related to ADLs [10]. Early mobilization is linked to reduced length of stay, improved functional mobility, and enhanced airway clearance. It is commonly recommended to address atelectasis, sputum retention, and postoperative complications [11]. Airway clearance techniques are utilized across various conditions, and early mobilization and ambulation are advocated to promote airway clearance and reduce postoperative complications [12]. This report aims to evaluate the effects of chest physical rehabilitation on reducing dyspnea and enhancing functional independence and quality of life (QOL) in patients with multilobar pneumonia.

## Case presentation

Patient information

A 65-year-old male presented with complaints of breathlessness, cough with whitish expectoration, and fever lasting for 14 days, prompting a visit to the hospital. Investigations, including sputum culture and X-ray, were conducted. The sputum culture indicated an infection, while the X-ray revealed multilobar pneumonia. The patient received medication, including an injection of Doxy 100 mg, an injection of ceftriaxone, and an injection of Hydrocort 50 mg. His other medical and surgical history was not significant. He was advised to undergo physical treatment for further care, and a tailored physical therapy protocol was initiated.

Clinical findings

Verbal consent was obtained from the patient before the assessment. The patient lay supine during the examination and was hemodynamically stable, conscious, and well-oriented to time, place, and people. According to the cardiorespiratory examination, the patient’s vital signs included a pulse rate of 80 beats per minute, a respiratory rate of 16 breaths per minute, and a blood pressure of 110/80 mmHg. Arterial blood gas findings revealed a pH of 7.41, PCO2 of 31.6 mmHg, PO2 of 67.2 mmHg, and HCO3 of 22 meq/L. An X-ray was performed, which showed increased bronchovesicular markings and bilateral heterogeneous opacities in the lower zones, suggesting multilobar pneumonia. Upon observation, chest symmetry was noted as symmetrical; however, chest movements were reduced. Auscultation revealed reduced air entry and the presence of crackles.

Investigations

**Figure 1:**
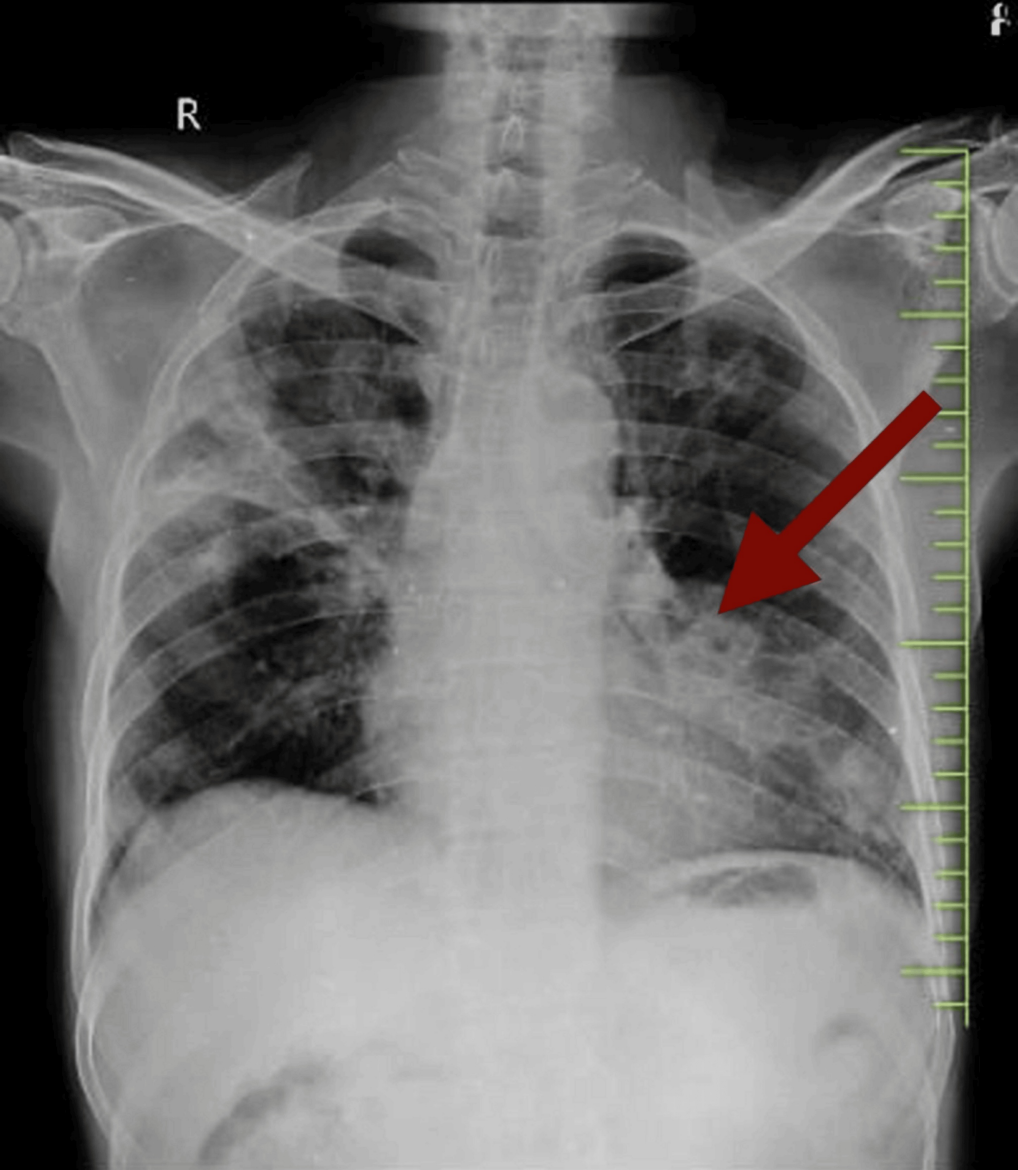
Chest X-ray showing increased bronchovesicular markings and bilateral heterogeneous opacities in lower zones

Chest X-ray findings are presented in Figure 1.

Timeline of events 

The timeline of events according to the patient’s condition is presented in Table 1.

**Table 1:** Timeline of events according to the patient’s condition

Events	Timeline
Date of admission	7/8/24
Date of assessment	9/8/24
Date of physiotherapy intervention	9/8/24
Date of discharge	15/8/24

Physiotherapy intervention 

Adjuvant therapies included supplemental oxygen, intravenous hydration, and chest physical therapy. Chest physiotherapy involved training in cough and breathing techniques, as well as positioning the patient to facilitate mucus drainage. Chest physical therapy comprised postural drainage, coughing, and vibrations to clear secretions. The interventions performed included forced expiration, bilateral upper and lower limb mobility, thoracic expansion exercises, and active breathing control. The details of these interventions are presented in Table 2.

**Table 2:** Physiotherapy interventions given to the patient QOL, quality of life

Sr no.	Problem list	Goals	Physiotherapy intervention	Rationale	Dosage
1	Lack of knowledge	Patient education	To explain the present condition and the advantages of a physical rehabilitation program to the patient	It facilitates the patient’s increased understanding of the condition. It helps the patient to have a good awareness of the illness in order to encourage active participation and increase the efficacy of treatment.	NA
2	Dyspnea	To reduce dyspnea	Dyspnea-relieving positions, pursed lip breathing; energy conservation technique	Aids in reducing the work of breathing, thus reducing dyspnea	10 reps × 1 set
3	Accumulation of secretions	To promote clearance of mucus and improve respiratory functions	Active cycle breathing technique; huffing and coughing	Helps to remove tracheobronchial secretions present in the airways. Additionally, they reduce airway resistance, which improves gas exchange and breathing	Five cycles
4	Reduced lung functioning	To improve lung functioning	Diaphragmatic breathing; thoracic expansion	Improves oxygenation and reduces respiratory muscle fatigue	10 reps × 1 set
5	Decreased strength and functional capacity	To improve upper limb strength	Strengthening of B/L upper and lower limb with 1 kg weight cuff/theraband and ambulation	Aid in improving peripheral muscle strength, hence improving functional capacity and cardiovascular endurance	10 reps × 1 set
6	Decreased functional mobility	To enhance functional mobility	Ambulation	One to two rounds daily (2-3 times) helps enhance the functional mobility of the patient	One to two rounds twice a day
7	Deconditioning	To improve the QOL of the patient	The home exercise regimen includes deep breathing exercises, mobility exercises for the upper and lower limbs, and ambulation	The home exercise regimen includes deep breathing exercises, mobility exercises for the upper and lower limbs, and ambulation	Twice a day

Outcome measures 

The Modified Medical Research Council (MMR) Dyspnea Scale, Functional Independence Scale, and QOL outcomes are presented in Table 3.

**Table 3:** Pre-intervention and post-intervention outcome measures assessed in the patient FIM, Functional Independence Measure; MMRC, Modified Medical Research Council; QOL, quality of life

Outcome measures	Pre-intervention	Post-intervention
MMRC Dyspnea Scale	Grade 2	Grade 1
FIM	86/126	100/126
QOL (SF-36)	45/100	68/100

## Discussion

This case study demonstrates that physical therapy is essential for the recovery of patients with pneumonia. The decrease in MMRC grades indicates a positive effect, as the patient can now perform daily tasks with less difficulty due to improved dyspnea control. There was also a significant improvement in Functional Independence Measure (FIM) scores across motor, cognitive, and functional independence domains. QOL scores, assessed via the SF-36 before and after treatment, further indicate that physical therapy positively impacts both the physical and mental health of patients. These improvements are particularly important as they correlate with enhanced performance in ADLs and an overall better QOL.

A similar study conducted by Liu et al. showed that physiotherapy plays a vital role in managing multilobular pneumonia, with treatment effectiveness evaluated using standardized outcome measures such as the MMRC scale, FIM, and QOL assessments. Pulmonary rehabilitation has been found to improve pulmonary function in patients suffering from severe pneumonia [13]. The exercise training program demonstrated notable benefits in functional capacity, dyspnea, and QOL [14]. Physiotherapy can enhance a patient’s QOL, oxygen saturation, and functional capacity while alleviating the severity of dyspnea. Chest physical therapy is a crucial therapeutic strategy for preventing pulmonary complications, and the patient's inspiratory muscle strength improved following this intervention. Research by Fikritama et al. revealed that effective coughing techniques and diaphragmatic breathing are chest physiotherapy treatments that can reduce dyspnea symptoms and enhance a patient’s QOL. They also indicated that patients with pneumonia benefit from the active cycle of breathing technique, which incorporates FET, thoracic expansion exercises, and a breathing control cycle [15]. Diaphragmatic breathing and thoracic expansion exercises were utilized to increase lung capacity. Early chest physiotherapy and mobilization - such as sitting up in bed or walking - can shorten hospital stays for patients with pneumonia [16]. Chest physical therapy, including nebulization, can help clear accumulated secretions. According to a study by Lestari et al., incorporating nebulization into the treatment protocol for patients suffering from pneumonia and atelectasis can prevent respiratory blockages due to excessive sputum production [17].

A study by Mertz and Johnstone suggested that early mobility is a fundamental intervention in the physiotherapy management of critically ill patients. Current guidelines for managing severe pneumonia emphasize mobilization alongside standard airway clearance procedures and continuous positive airway pressure. Recommendations include at least 20 minutes of sitting outside of bed within the first 24 hours of admission [18].

Fink showed that techniques such as directed coughing, FET, active breathing cycle, and autogenic drainage have been developed to maximize expiratory flow and facilitate airway clearance [19]. Additionally, a study by Andrian and Rosyid highlighted that pursed lip breathing therapy can assist in pushing secretions during expiration and increase alveolar pressure, promoting lung expansion. This nonpharmacological treatment for dyspnea can also enhance oxygen saturation and decrease respiratory rate [20].

## Conclusions

The highlighted case demonstrates that early intervention is essential for reducing dyspnea and improving physical function during the acute phase of pneumonia. The prompt initiation of chest physical therapy is crucial for clearing excess secretions and facilitating airway clearance as part of the recovery phase. Enhancing the patient’s respiratory condition and accelerating recovery were the cornerstones of the treatment protocol. Improved airway clearance offers two key advantages: enhanced gas exchange and reduced respiratory effort. Consequently, the patient experienced significant improvements in dyspnea, functional independence, and overall QOL as a result of the tailored pulmonary rehabilitation.
